# Transcriptomic analysis of the harvested endothelial cells in a swine model of mechanical thrombectomy

**DOI:** 10.1007/s00234-018-2033-1

**Published:** 2018-05-14

**Authors:** Nasren Jaff, Rikard Grankvist, Lars Muhl, Arvin Chireh, Mikael Sandell, Stefan Jonsson, Fabian Arnberg, Ulf Eriksson, Staffan Holmin

**Affiliations:** 1 Department of Clinical Neuroscience, Karolinska Institutet, SE-171 76 Stockholm, Sweden; 2 Department of Neuroradiology, Karolinska University Hospital, Solna, SE-171 76 Stockholm, Sweden; 3 Department of Medical Biochemistry and Biophysics (MBB), Karolinska Institutet, Stockholm, Sweden; 4 Department of Materials Science, Royal Institute of Technology, Stockholm, Sweden

**Keywords:** Endothelial cells, Transcriptomic analysis, Thrombectomy, Cell culture

## Abstract

**Purpose:**

In mechanical thrombectomy (MT) for ischemic stroke, endothelial cells (ECs) from intracranial blood vessels adhere to the stent retriever device and can be harvested. However, understanding the molecular biology and the role of the endothelium in different pathological conditions remains insufficient. The purpose of the study was to characterize and analyze the molecular aspect of harvested ECs using cell culture and transcriptomic techniques in an MT swine model relevant to clinical ischemic stroke.

**Methods:**

In swine, preformed thrombi were injected into the external carotid and subclavian arteries to occlude their branches. MT was performed according to clinical routine. The stent retriever device and thrombus were treated with cell dissociation buffer. The resulting cell suspension was analyzed by immunohistochemistry and was cultured. Cultured cells were analyzed using single-cell RNA sequencing (scRNA-seq) after fluorescence-activated cell sorting (FACS).

**Results:**

A total number of 37 samples were obtained containing CD31-positive cells. Cell culture was successful in 90% of samples, and the cells expressed multiple typical EC protein markers. Eighty-nine percent of the sorted cells yielded high-quality transcriptomes, and single-cell transcriptomes from cultured cells showed that they expressed typical endothelial gene patterns. Gene expression analysis of ECs from an occluded artery did not show distinctive clustering into subtypes.

**Conclusion:**

ECs harvested during MT can be cultured and analyzed using single-cell transcriptomic techniques. This analysis can be implemented in clinical practice to study the EC gene expression of comorbidities, such as hypertension, diabetes mellitus, and metabolic syndrome, in patients suffering from acute ischemic stroke.

## Introduction

Endothelial cells (ECs) play important roles in many aspects of vascular biology and are thereby involved in the pathophysiological processes of several diseases. In addition to its role as a selective permeability barrier, the ECs are unique multifunctional cells with critical metabolic and synthetic functions [1]. Impaired endothelial function may cause thrombosis and altered glucose and fatty acid transport, which is seen in patients with ischemic heart disease, diabetes, hypertension, and hypercholesterolemia [1–6]. Many of these processes contribute to the pathology of ischemic stroke. Great importance has been suggested for ECs in the activation and propagation of vascular diseases [7].

The advent of mechanical thrombectomy to treat acute ischemic stroke (AIS) [8–12] has enabled unprecedented access to thrombotic material [13–15]. Recently, an adapted new method of harvesting EC biopsy in AIS was demonstrated [16]. We hypothesized that applicability of this protocol for research would benefit from a molecular biology analysis with next-generation sequencing transcriptomics. To maximize the yield of gene expression information from the analyzed cells, we aimed to use single-cell RNA-seq (scRNA-seq) to enable comprehensive transcriptomic analysis. This study presents an effective method for collecting endothelial cells in a swine model of MT using standard clinical devices, culturing them, and subsequently performing single-cell RNA-seq.

## Materials and methods

### Animal preparation

All procedures were conducted according to the Karolinska Institute guidelines for experiments on large animals and were approved by the ethics committee (Norra Stockholms Djurförsöksetiska nämnd, N87/15 and N162/16). Fourteen female Yorkshire-Swedish farm swine ranging from 30 to 35 kg were used in this study. The procedures were conducted with the swine under general anesthesia by sodium pentobarbital and mechanical ventilation. The animals were euthanized after the experiments.

### Thrombus preparation, angiography, and thrombus injection

We prepared the blood clot for a selective thromboembolization by mixing swine whole blood with a commercial hemostasis agent containing human fibrinogen, factor VIII, human thrombin, calcium chloride, and synthetic aprotinin (TISSEEL, Baxter, IL, USA). The mixture was observed until adequate clot formation had occurred, and elongated thrombi were prepared using scalpel dissection.

A 6F guiding catheter (Cordis, CA, USA) sheath was inserted into the right common femoral artery and continuously flushed with saline. External carotid artery angiography was performed. For selective thromboembolization, the preformed thrombus was injected into a 6F guide catheter with the tip placed in the external carotid artery or the subclavian artery. After connecting the silicone tube containing a prepared thrombus into the guide catheter, we injected (20 ml) of saline into the tube to force the thrombus into the catheter. After an angiogram had been obtained to document the occlusion, the guide catheter was retrieved. To simulate the clinical situation in ischemic stroke, the occlusion was maintained at durations 0, 5, 3, or 6 h before thrombectomy was performed.

### Harvesting and isolation of cells

MT was carried out via standard femoral access by using a 6F Envoy guiding catheter (Cordis, USA). The catheter was navigated under fluoroscopy guidance to the external carotid artery (ECA) and subclavian artery (SCA) territories. A microcatheter (Prowler Select Plus, Cordis, USA) was navigated to arterial branches, and once in the right position in arteries with inner lumen diameter of 2–4 mm, the Solitaire 6 × 30 mm device (Covidien-Medtronic, Ireland) was used as in clinical practice, including leaving the device for 5 min to expand before retrieval. The device was resheathed into the microcatheter until the beginning of the thrombus before retraction of the whole system. During retrieval, aspiration through the guide catheter was performed according to clinical routine. Directly after removing the device from the guide catheter lumen, the device was immersed and cleaned in dissociation buffer (0.5% BSA, 100 μg/l heparin, 2 mM EDTA in PBS), to detach possible EC from the device. Using the same device, five attempts were made in different vessels. Each successful attempt resulted in one sample.

### Sample handling and preparation procedure

The samples were centrifuged at 300*g*, the supernatant was removed, and then erythrocyte-lysing buffer was added and the samples were centrifuged again at 300*g*. Positive control samples were obtained from swine (*n* = 2), by mechanically scratching the endothelial lining of a surgically dissected artery. The control cells were processed in the same way as described above.

### Cell culture

The tubes were incubated for 5 min on ice with agitation of tubes once every minute. Five milliliters of PBS was added to each tube and centrifuged for 7 min at 300*g*. The supernatant was discarded and 3 ml of culture medium was added. The primary EC cells were cultured using gelatin-coated cell culture plates, 1% *w*/*v* in PBS (Sigma-Aldrich, Schnelldorf, Germany) and endothelial cell growth medium micro-vascular 2 (EGMV2) (PromoCell, Germany). The culture plates contained growth supplements, 10 U/ml penicillin, 10 μg/ml streptomycin, and 25 μg/ml amphotericin B (Thermo Fisher Scientific Inc., CA, USA). Two to three days after initial culture, the remaining thrombus and nonadhered cells were washed away with EGMV2 medium. Thereafter, the medium was replaced at intervals of 3 to 4 days, and the cells were passaged when near confluence, using 0.05% trypsin/EDTA (Thermo Fisher Scientific, CA, USA).

### Immunohistochemistry

Primary isolated cells were cultured on gelatin-coated glass coverslips in 6-well cell culture plates, in EGMV2. Cells were fixed with ice-cold 4% paraformaldehyde (PFA) in PBS for 10 min at room temperature (RT). After fixation, cells were blocked with serum-free protein blocking solution (DAKO, Glostrup, Denmark), supplemented with 0.2% Triton X-100 (Sigma-Aldrich) for > 1 h at RT. Thereafter, cells were sequentially incubated with primary mouse anti-pig-CD31 dil. 1:100 (AbD Serotec, Germany), rabbit anti-vWF dil. 1:200 (DAKO), and goat anti-VE-cadherin dil. 1:300 (Santa Cruz Biotechnology Inc.) for 24 h at 4 °C. The cells were rinsed in PBS and subsequently incubated with species-specific secondary antibodies diluted in PBS: Alexa Fluor 488 donkey anti-mouse, Alexa Fluor 555 donkey anti-rabbit, Alexa Fluor 647 donkey anti-goat, all dil. 1:500 (Molecular Probes/Invitrogen, Germany), and Cy3 goat anti-mouse (dil. 1:1000, Jackson Immunoresearch, Baltimore, USA). Cells were mounted with ProLong Gold mounting medium containing DAPI (Molecular Probes, CA, USA). Evaluation of the staining and image capture of immunofluorescence micrographs was carried out by using an upright laser scanning, confocal microscope (LSM 700, Carl Zeiss GmbH, Göttingen, Germany).

### Single-cell isolation by FACS and cDNA library preparation

To perform single-cell scRNA-seq, we sorted individual cultured EC onto a 384-well plate preloaded with lysis buffer using fluorescence-activated cell sorting (FACS). A set of cultured endothelial cells from MT and a surgically excised control vessel was prepared for this purpose. The cells were first centrifuged and resuspended in FACS buffer (1× PBS, 4 ml of 0.5 M EDTA and 5 g of BSA). The cells were incubated with an antibody cocktail (CD31, CD146, DAPI) for 30 min at 4 °C, washed with FACS buffer, and resuspended in 500 μl FACS buffer for single-cell sorting. The cells were sorted with the BD FACSJazz cell sorter (BD Biosciences, Franklin Lakes, NJ, USA). Single cells were sorted one per well onto a 384-well plate prepared with lysis buffer. After sorting, the plates were immediately stored at −80 °C and processed for sequencing at a later stage.

scRNA-seq was performed according to the Smart-seq2 protocol [17]. The Illumina HiSeq system (Illumina, San Diego, CA, USA) was used to sequence with a read length of 1 × 50 base pairs (single-read).

### Data processing and analysis

Sequencing data was analyzed using an established workflow [18]. Reads were mapped to the reference genome Sscrofa10.2. Quality control was performed before and after mapping, with FastQC version 0.11.5 and QualiMap version 2.2, respectively. Gene expression values were normalized and expressed as log_2_ counts per million (CPM). Highly variable genes were selected for principal component analysis (PCA) and unsupervised hierarchical clustering. Cell cycle-dependent effects were mitigated by using ccRemover [19]. DESeq2 package from Bioconductor for R (version 3.4, https://bioconductor.org/packages/release/bioc/html/DESeq2.html) was used for differential expression analysis on a subset of genes related to EC physiology. For the differential expression analysis, an absolute log_2_ fold change threshold of ≥ 1 was used as threshold.

### Scanning electron microscopy of stent retriever device

In order to detect any corrosion attacks on the stent by the dissociation buffer, we investigated the Solitaire stent retriever (Covidien-Medtronic, Ireland) in a light optical microscopy (LOM). The detachment/attachment zone was studied by scanning electron microscopy (SEM), before and after soaking in a dissociated buffer at room temperature. The chemical compositions of the detachment/attachment zone and its surroundings were determined by energy-dispersive X-ray spectroscopy in the SEM. In addition, the chemical compositions of precipitates after soaking were analyzed.

## Results

### Mechanical thrombectomy and endothelial cell harvesting

**Fig. 1:**
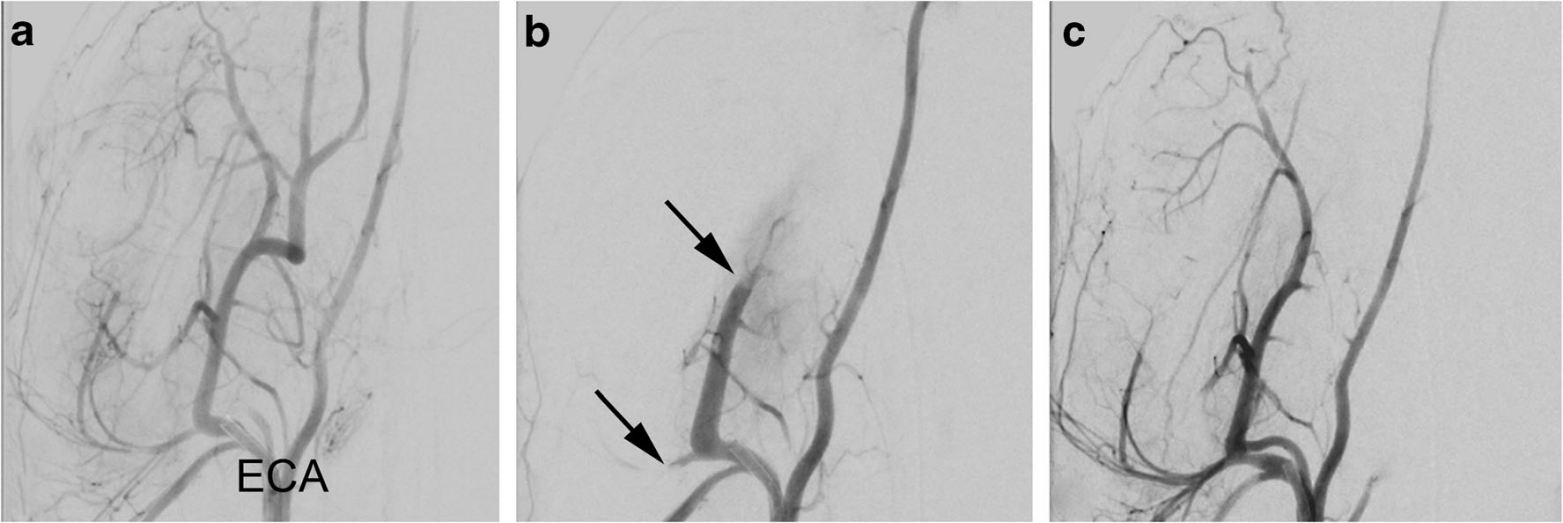
Anteroposterior angiographies of mechanical thrombectomy procedure. **a** Catheter tip in the right external carotid artery (ECA) before thrombus injection, showing patent circulation. **b** After a thrombus injection, a large and a smaller segments are occluded (arrows). **c** After two passes of mechanical thrombectomy of the occluded segments using a stent retriever device, the smaller segment and the base of the larger segment are again patent. More distal occlusions to the large segment remain

The procedure was carried out in 14 swine, and the retrievals were effective in all target vessels. The devices were successfully navigated to different branches within the external carotid artery and subclavian artery territory without technical problems (Fig. 1). We were able to harvest viable cells from both occluded and nonoccluded arteries.

### Expansion and proliferation of endothelial cells from mechanical thrombectomy

**Fig. 2:**
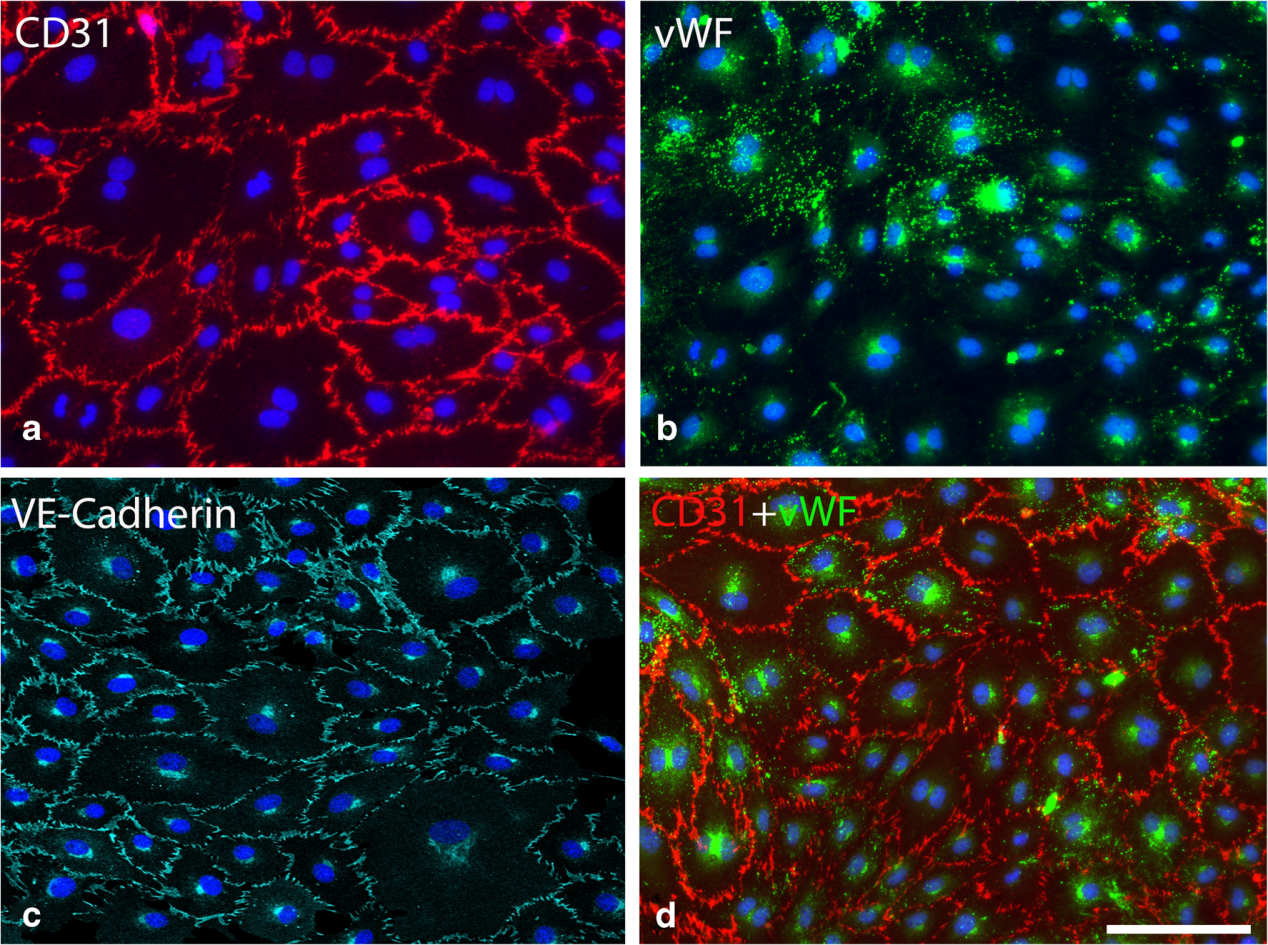
Immunohistochemistry staining of endothelial cells from cell culture. Endothelial cells revealed by positive CD31 membrane staining (**a**), vWF cytosol staining (**b**), VE-cadherin staining (**c**), and positive CD31 membrane staining and vWF cytosol staining (**d**). Scale bars = 100 μm

We next analyzed whether we could isolate enough viable endothelial cells from MT to expand the cells in culture. Samples (*n* = 37) from the 14 swine were successfully isolated and cultured. In 80% of the samples, the cells showed excellent growth capacity and could be passaged for up to ten times. Endothelial cell identities were verified by immunohistochemistry. The cells showed CD31, vWF, and VE-cadherin immunoreactivity (Fig. 2a-c). Subsets of cells also showed double-positive staining for vWF and CD31 and were considered true phenotypic ECs (Fig. 2d).

### Single-cell RNA sequencing of cultured endothelial cells

**Fig. 3:**
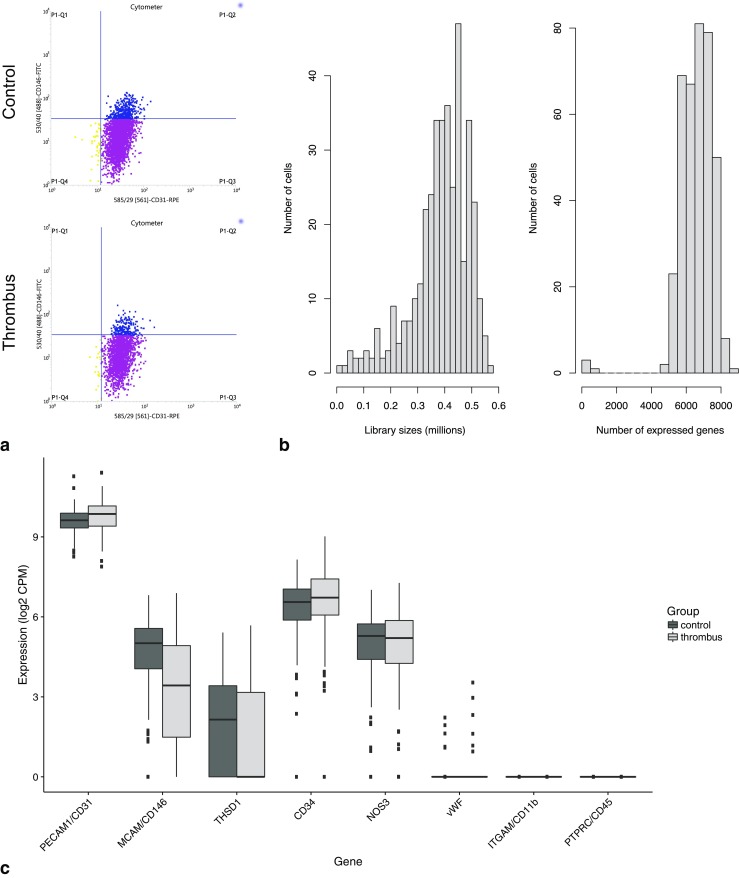
**a** FACS final gates for EC cell sorting. CD31+ and CD146+ ECs were sorted for single-cell RNA sequencing. **b** Histograms of counts and number of expressed genes over all cells. **c** Expression of gene characteristics of endothelial cells in thrombus and nonthrombus samples as well as genes that used as a negative controls (CD-11b and CD-45). Differential expression assessed by DESeq2, showing no significant difference between selected genes with a cutoff of absolute log_2_ fold change ≥ 1 (*p* > 0.05)

**Fig. 4:**
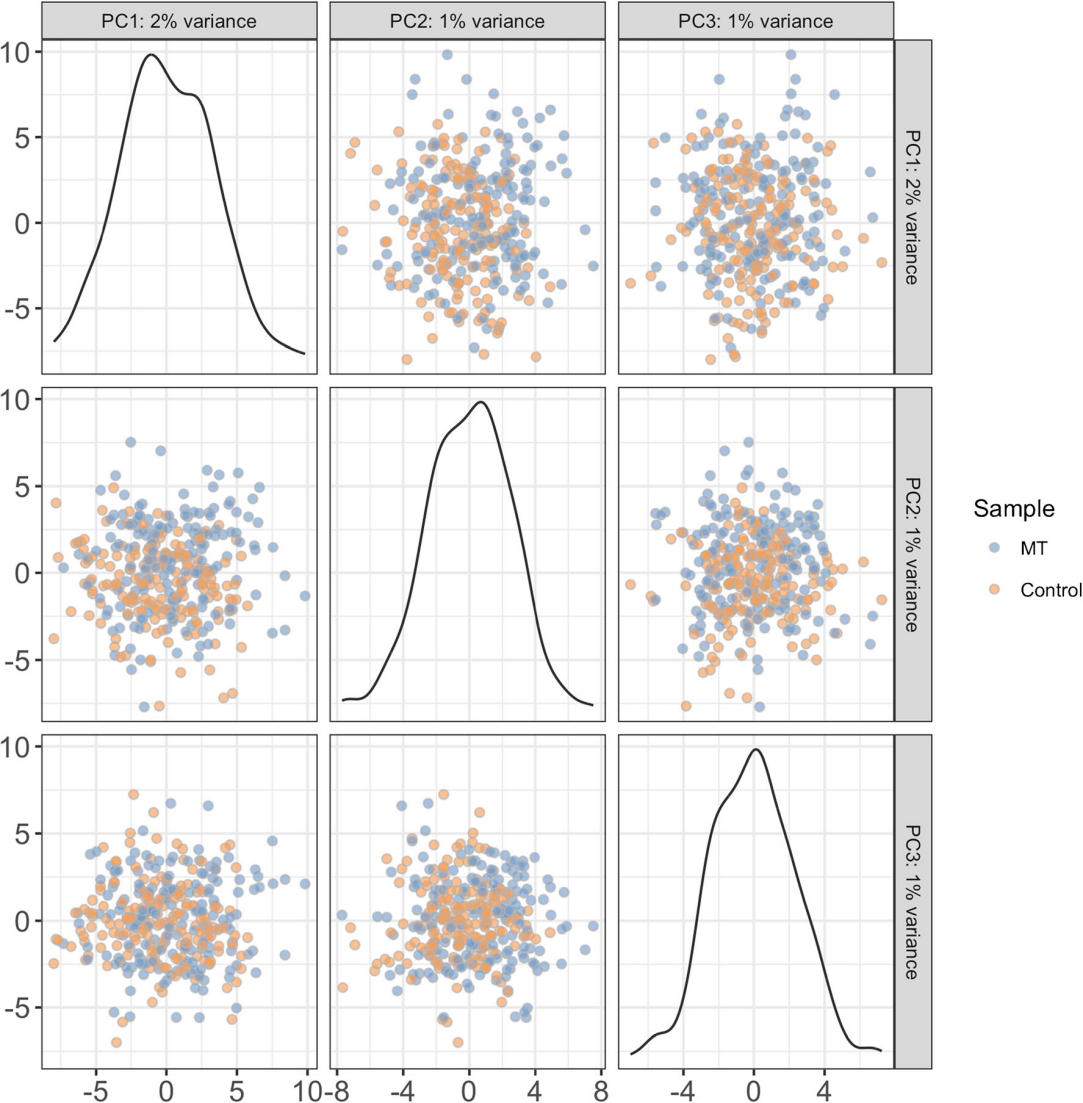
**a** Principal component analyses (PCA) plot, showing the first 3 PCA components

**Fig. 5:**
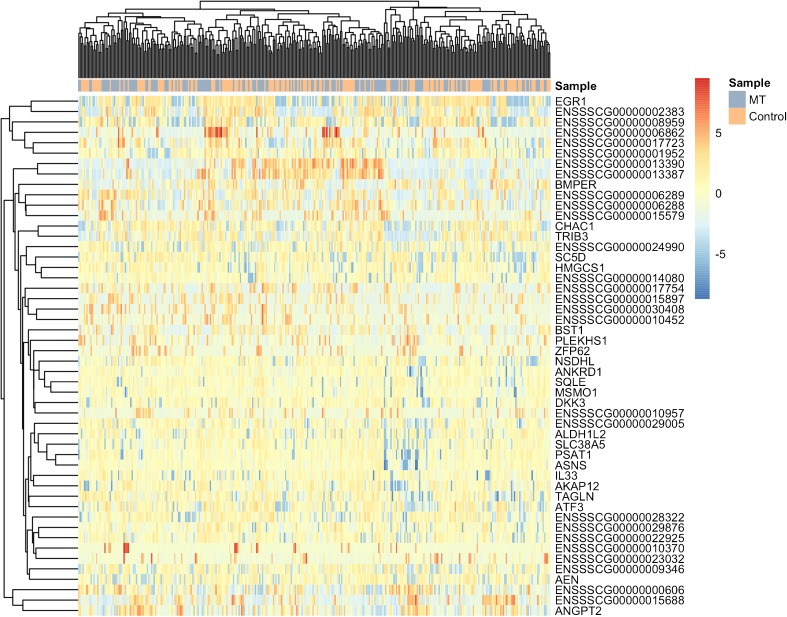
Heatmap for the 50 high variable genes (HVG). Rows are genes; columns are cells. Clustering is done based on Euclidean distance. No clear clustering of cells within or between mechanical thrombectomy (MT) and control is seen

To analyze the functional heterogeneity and to identify the different phenotypes of ECs, we used single-cell RNA-seq on a subset of cultured cells from cells obtained during the MT procedure. A total of 384 ECs were collected by FACS, out of which 345 (90%) showed high-quality metrics (Fig. 3). Thirty-nine cells were excluded for failing one or more of the following quality criteria: limited sequence depth (*n* = 33), few expressed genes (*n* = 4), too large proportion of mitochondrial genes (*n* = 9), and too large proportion of ERCC sequences (*n* = 8). The cutoff level for each of the quality metrics was ≥ 3 median absolute deviations. One hundred seventy-five cells of the control samples (91%) and 170 cells of the MT samples (89%) were selected for further analysis. Of the 25,414 annotated genes in pig genome, 10,695 genes had an average count of more than 0.5 log_2_ CPM in the high-quality samples. Three thousand two hundred sixty-four genes had a significant variance (*p* < 0.05) and an estimated biological variance larger than 0.5. The results showed high sequencing quality in cells both from control arteries not containing thrombus and those containing thrombus (Fig. 3b). Genes with known upregulation or downregulation in endothelial cells, such as PECAM (CD31), MCAM (CD146), CD34, and vWF, were selected for targeted analysis. The pattern was generally consistent with typical EC expression in both groups. None of the genes were significantly different between the groups using adjusted *p* < 0.05 (false discovery rate = 0.1) and absolute log_2_ fold change > 1 as criteria (Fig. 3c). After controlling for cell cycle, principal component analysis (PCA) and heatmaps for highly variable genes (HVG) did not show any distinct clustering within or between thrombectomy and control samples (Fig. 4 and Fig. 5).

### Structural analysis of stent retriever device

To evaluate the safety of the sampling protocol in a potential clinical application with repeated MT passages involving dissociation buffer, we examined the Solitaire stent retriever (Covidien-Medtronic, Ireland) after the procedure for signs of damage. Visual inspection showed no apparent structural damage even after multiple uses. Further analysis by in vitro exposition of the stent to the cell dissociation buffer and subsequent LOM and SEM showed no structural damage and no signs of corrosion attack or other surface effects on the device other than some saline precipitation (data not shown).

## Discussion

Endothelial tissue availability has been a major constraint when investigating the cellular mechanism of vascular diseases or diseases affected by vascular dysfunction. The critical step in bridging the gap between clinical and basic science is a suitable and safe method to sample ECs and to develop a protocol that enables genetic transcription analysis. In this study, we used a swine model to characterize the molecular biology of the ECs harvested during MT. To achieve high-quality transcriptomes, we needed an intermediate step of cell culture. We demonstrate that it is possible to, by using the routine MT protocol, sample, culture the cells and, then analyze the gene expression in regional cultured ECs using transcriptomics.

Cell culture allows direct characterization of endothelial phenotype and is useful for understanding the interaction between ECs and various mediators; however, it is often criticized due to the uncertain relevance of results to the in vivo situation in humans [20]. The culture step is known to cause changes in gene expression, especially if many passages are performed before analysis. In the present study, the cell culture probably caused changes in the transcriptome of the endothelial cells; thereby, clues to pathophysiological dysfunction of endothelial cell transcriptomes in the acute phase of vessel occlusion may have been lost. Sun et al. demonstrated that they were able to directly perform qPCR on ECs harvested by inserting a curved stainless steel guide wire into the iliac artery in patients undergoing routine catheter angiography [21]. We were not able to reproduce this strategy in this model of MT in small arteries since our attempts to take the cells directly from MT to FACS and scRNA-seq failed. A possible reason for this could be compromised cell viability due to thrombus effect on the endothelial cells or by the relatively “dirty” cell mix that is obtained in the setting of MT. Complete manual removal of the thrombus from the stent retriever before EC dissociation, as described in the human setting by Sheth et al. [16], could possibly resolve this issue.

The limited numbers of the collected ECs in the setting of MT currently seem to limit materials for molecular analysis such as single-cell mRNA transcriptomics on freshly obtained cells. The intermediate step of cell culture to expand the ECs for transcriptomic analysis implies that the protocol in its current form is best suitable for studying chronic comorbidities, such as diabetes mellitus or hypertension, associated with ischemic stroke. Furthermore, the approach also shows the feasibility of creating an EC-in-AIS biobank.

Single-cell mRNA sequencing has emerged as a powerful method to simultaneously measure cell-to-cell expression variability of thousands of genes [22], and such analysis is possible with large amount of input cells. Sun et al. collected peripheral ECs, sorted them using FACS, and analyzed single-cell gene expression. While Sun et al. yielded good results using quantitative RT-PCR on high-throughput microfluidic platform [21, 23, 24], there is an inherent limitation in the scope of analysis that can be performed on data obtained using specific primers in RT-PCR in comparison to scRNA-seq. scRNA-seq offers considerably more expression information and is amenable to exploratory data analysis and hypothesis generation, to elucidate the role of ECs in neurovascular pathology.

An important possibility in transcriptomic analysis is the identification of subtypes within an input cell type, in this case ECs. When looking at differential expression of EC markers (positive and negative markers) in the thrombus compared to the control sample, none of the genes showed significant difference, suggesting a similar expression pattern between groups in regard to EC markers. Initial analysis showed some apparent clustering on PCA hierarchical clustering and heatmap analysis of highly variable genes (not shown). We considered cell cycle state as a source of bias in this case, as this was not controlled for in the sorting stage. Furthermore, the genes with large biological variance are almost exclusively genes that are associated with cell cycle-dependent variability. Therefore, we used a data processing method, ccRemover [19], to identify and remove principal components likely caused by cell cycle variation among the cells. Subsequent PCA and heatmaps did not show apparent clustering either within or between samples. If there are indeed phenotypical subtypes of ECs in the swine ECA, these phenotypes were not apparent in our sample after cell culture.

## Conclusions

Evaluation of intracranial vascular endothelial dysfunction in patients undergoing ischemic stroke or other endovascular diseases has been limited so far. In this study, we present a pipeline for harvesting, culturing, and then analyzing endothelial cells using FACS and scRNA-seq in a model of mechanical thrombectomy. This pipeline for analysis could readily be integrated with the method of EC harvest in human AIS recently reported by Sheth et al. [16]. The time of the procedure is not extended, and the device itself is not affected by the harvesting method. This method allows systematic population of a biobank of human brain endothelial cell transcriptomes from patients undergoing ischemic stroke, providing a wealth of new research material to investigate the cellular and molecular mechanisms by which the vascular endothelium mediates the processes of physiology and pathophysiology of stroke and associated comorbidities.

## Abbreviations

MT: Mechanical thrombectomy
scRNA-seq: Single-cell RNA sequencing
EC: Endothelial cell
FACS: Fluorescence-activated cell sorting
AIS: Acute ischemic stroke
ECA: External carotid artery
SCA: Subclavian artery
PBS: Phosphate-buffered saline
PAF: Paraformaldehyde
PCA: Principal component analysis
LOM: Light optical microscopy
SEM: Scanning electron microscopy
